# From Lab to Workplace: Efficacy of Skin Protection Creams Against Hydrophobic Working Materials

**DOI:** 10.3390/jcm14238470

**Published:** 2025-11-28

**Authors:** Anja Dick, Magdalena Metzger, Peter Dungel

**Affiliations:** 1 Peter Greven Physioderm GmbH, 53881 Euskirchen, Germany; 2 Ludwig Boltzmann Institute for Traumatology, The Research Center in Cooperation with AUVA, 1200 Vienna, Austria; magdalena.metzger@trauma.lbg.ac.at (M.M.); peter.dungel@trauma.lbg.ac.at (P.D.)

**Keywords:** contact dermatitis, irritant, occupation, prevention, skin

## Abstract

Occupational dermatoses represent a significant challenge across numerous industries. Therefore, occupational skin protection creams are frequently used as a preventative measure, yet their efficacy, particularly against hydrophobic working materials, remains inconclusive. This review provides an overview of the current knowledge concerning the mechanisms by which skin protection creams support skin barrier function, as well as the limitations of available testing methods. Current evaluation methods range from in vitro assays to ex vivo and in vivo models, each possessing distinct advantages and disadvantages. While several clinical studies demonstrate effectiveness against hydrophilic irritants, standardized and ethically viable methods for assessing protection against hydrophobic substances are lacking. Future research should focus on the development and validation of improved in vitro and ex vivo models, coupled with enhanced workplace simulation techniques, to facilitate a more accurate translation of laboratory findings to real-world occupational settings. Consistent and reliable testing is essential to ensure continued efficacy of these products in light of evolving regulatory landscapes.

## 1. Introduction

Contact dermatitis is the most common occupational skin disease, characterized by acute or chronic inflammation of the skin resulting from exposure to chemical or physical substances. It is generally classified into two types: irritant contact dermatitis (ICD) and allergic contact dermatitis (ACD). ICD accounts for approximately 90% of cases and typically develops from repeated exposure to irritants. It arises due to direct damage to epidermal cells, which triggers an innate immune response and leads to inflammatory skin reactions. In contrast, ACD is mediated by a delayed Type IV hypersensitivity reaction of the immune system to specific allergens [1]. The most prevalent form of ICD observed in clinical settings is the chronic type, which develops after prolonged, repeated exposure to mild or borderline irritants. It commonly presents as dry, dull, erythematous, and scaly skin, often accompanied by lichenification [2]. ICD frequently affects young adults and is strongly associated with occupational exposure, although it can also occur in non-occupational contexts [3].

Approximately 80% of occupational hand eczema cases are concentrated within seven occupational settings: hairdressing, metalworking, healthcare, food industry, construction, painting, and cleaning. Occupational hand eczema in these settings is caused by various irritants. In general, irritants may be chemical or physical agents capable of inducing cellular damage following prolonged or high-concentration exposure. In high-risk occupations—such as hairdressing, healthcare, or cleaning—key irritants include water, frequent exposure to wet environments (wet work), and water-soluble chemicals such as detergents, surfactants, acids, and alkalis [4,5]. However, lipophilic substances should not be overlooked. Solvents or oils (e.g., cutting oils and machining fluids in metalworking) are also significant risk factors for ICD development [5,6,7].

Once a specific irritant has been identified, preventive measures should be implemented to reduce future exposure. These include the use of personal protective equipment (PPE) such as gloves as well as the application of skin protection creams. Skin protection creams are widely used in occupational settings. Yet, a major knowledge gap remains: their efficacy against hydrophobic irritants (e.g., oils, solvents, and other hydrophobic materials) has not been systematically studied under standardized and ethically feasible conditions. This review aims to summarize current knowledge regarding the mechanisms and testing of skin protection creams formulated to protect the skin against hydrophobic working materials. It also addresses the ethical and methodological challenges associated with their evaluation.

## 2. Occupational Skin Protection Creams Against Hydrophobic Working Materials

**Figure 1:**
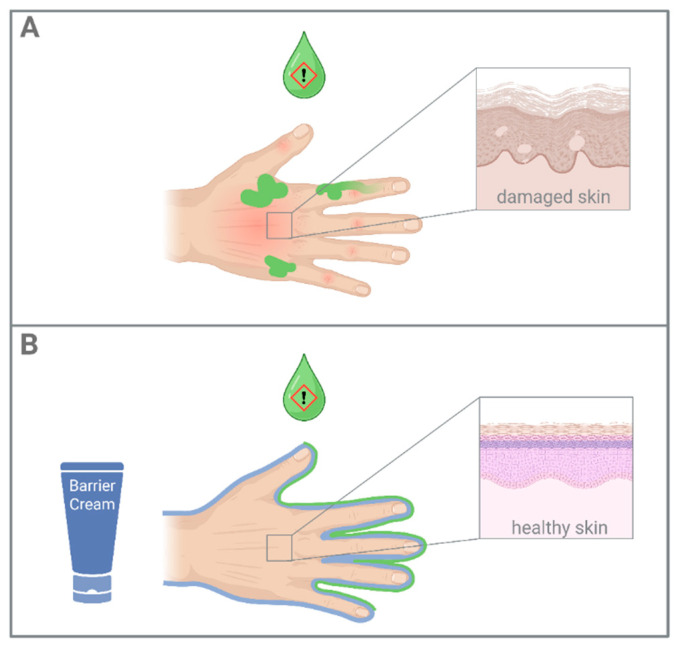
Schematic showing the effect of barrier creams on skin protection. Panel (**A**) depicts damaged skin, while Panel (**B**) shows healthy skin protected by barrier cream.

Occupational skin protection generally includes skin protection, skin care and skin cleansing products. Together, these products play a crucial role in the prevention and management of occupational hand eczema [8]. Skin protection creams are specifically designed to shield the skin from low-grade irritants that are non-toxic, non-carcinogenic, and non-sensitizing—such as water, detergents, and cutting fluids (Figure 1). While they do not replace primary protective measures such as gloves, they serve as a valuable alternative in situations where glove use is impractical due to safety concerns or restricted dexterity [9].

Skin protection creams are generally classified into two categories based on their function: pre-work barrier creams (BCs) and after-work emollients. However, the effectiveness of BCs—particularly against hydrophobic working materials—remains controversial and is not universally accepted [10,11,12]. Nonetheless, the combined use of both types has been shown to yield superior outcomes in the prevention of occupational dermatitis [13].

### 2.1. Barrier Creams

Barrier creams (BCs) are recommended for application prior to exposure to skin-irritating substances (pre-work), as well as after each work break or at defined intervals (e.g., midway through a work shift). Prior to reapplication, the skin should be thoroughly cleansed and dried to minimize the risk of increased absorption of residual irritants. The primary function of BCs is to protect the skin by reducing the absorption and penetration of potential irritants and allergens. This protective effect is intended to lower the risk of developing ICD and ACD in both occupational and non-occupational settings [8,9].

Although the exact mechanism of action of BCs is not fully understood, they are generally believed to function as physical barriers that impede the penetration of harmful substances. Lipophilic and lipophobic formulations are thought to provide protection against hydrophilic and hydrophobic agents, respectively. For example, BCs targeting protection from hydrophobic irritants—such as oils, varnishes, and organic solvents—typically contain hydrophilic (lipophobic) components, including propylene glycol, glycerin, and sorbitol [12,14].

BCs may also contain other functional components, including astringents (e.g., tannins), absorbers (e.g., zinc oxide, talc), and chelating agents. Zinc oxide, for instance, provides a shielding effect, while tannins promote skin hardening, thereby reducing mechanical trauma to the skin barrier. Conversely, ingredients known to enhance skin penetration, such as urea, are considered unsuitable for inclusion in BC formulations [10]. Key functional ingredients commonly used in BCs are summarized in Table 1.

### 2.2. After-Work Emollients

After-work emollients do not provide direct protection against irritants and are therefore not categorized based on specific irritants or occupational substances. Instead, their primary function is to support the regeneration and reinforcement of the skin’s barrier. Beyond shielding the body from external aggressors, the skin barrier plays a critical role in preventing transepidermal water loss (TEWL). The Stratum corneum (SC), the outermost layer of the epidermis, serves as the first line of defense against environmental stressors, including irritants. The barrier function of the SC depends largely on its lipid composition (e.g., ceramides, cholesterol, and free fatty acids) as well as its water content [15,16,17]. However, the arrangement of the lipids between the corneocytes as a lamellar structure is crucial for the barrier function of the skin [16]. Exposure to hydrophobic irritants, such as organic solvents, leads to the depletion of lipids surrounding the corneocytes. This disruption results in increased TEWL and heightened skin permeability. Consequently, proinflammatory cytokines are released, initiating inflammatory responses that may culminate in ICD [1].

After-work emollients are specifically designed to support skin barrier restoration through multiple mechanisms, including hydration via moisturizing substances and topical replenishment of physiological lipids [17,18]. Glycerol-based formulations, for example, are commonly used to enhance skin moisturization. Unlike BCs, urea can also be used as a moisturizing substance in after-work emollients. Common functional ingredients used in after-work emollients are shown in Table 1. In addition to the ingredients themselves, the type of formulation is a decisive factor for the effectiveness of after-work emollients. Because of the lamellar structure of skin lipids, multilamellar emollient formulations integrate more easily into the lipid matrix and thereby support barrier regeneration [18,19].

**Table 1:** Key functional ingredients and their mechanisms commonly used in barrier and emollient creams.

Functional Class	Examples (INCI Names)	Mechanisms	Ref.
**barrier creams:**			
film formers	PERFLUORODECALIN PVP	Create a thin protective, often occlusive or semi-occlusive layer on the skin to prevent moisture loss and shield the skin from environmental stressors.	[10,20,21]
absorbers	TALC KAOLIN	Form a dry, powdery layer that helps to prevent oils and other hydrophobic materials from adhering directly to the skin through absorption.	[10,20]
astringents	HAMAMELIS VIRGINIA EXTRACT ALUMINUM CHLOROHYDRATE	Contract skin tissue, and reduce perspiration and oil production. May also reduce inflammation.	[10,12,22]
chelating agents	DISODIUM EDTA	Bind to metal ions (e.g., calcium, magnesium) to reduce oxidative damage.	[10,23]
**after-work emollients:**			
moisturizers	GLYCERIN UREA	Draw water into the stratum corneum, improving hydration and maintaining skin elasticity.	[22]
lipids	CERAMIDES LINOLEIC ACID CHOLESTEROL	Restore skin’s lipid barrier, enhancing hydration, skin flexibility, and protection against environmental damage.	[18]

Due to these properties, regular application of after-work emollients is considered essential at the end of the workday and following any skin cleansing procedures [8].

## 3. Efficacy and Clinical Evidence

For years, several approaches have been developed to assess the effectiveness of skin protection products against established irritants. The evaluation of skin protection creams involves in vitro, ex vivo, and in vivo models. Each approach offers distinct advantages and limitations, particularly concerning the ethical and practical constraints in assessing exposure to harmful substances. Test results obtained from in vitro methods, e.g., glass slide test or filter paper membrane tests are considered to have only indicative value. Also studies on contact-angle and wettability methods can provide insights into film forming behavior on skin [24]. These methods reveal certain aspects of protective effects, but they cannot replicate the physiological processes in vivo. Furthermore, these in vitro methods have not yet been correlated with in vivo models, which means their reliability for assessing the efficacy of skin protection products remains very limited. In vitro models such as keratinocyte cultures, three-dimensional human skin models or ex vivo models using animal or human tissue such as the perfused bovine udder model allow the examination of biochemical, immunological, and/or morphological parameters. These models can provide indirect information about the expected efficacy and are therefore of greater significance. However, these are methods with mostly short-term/one-time exposure to noxious substances. Therefore, they are not suitable for drawing conclusions about a possible protective effect in the case of cumulative exposure to irritants. Cohort and intervention studies are considered the gold standard for demonstrating effectiveness, as they reflect real workplace conditions. If they are not feasible, evaluation using in vivo methods, such as the repetitive irritation model are recommended [8,11].

Due to the wide range of occupational irritants and the ethical and methodological limitations in testing many of them, standard substances—such as sodium lauryl sulfate or lactic acid—are commonly used as representative irritants. However, suitable standard irritants for hydrophobic substances remain scarce. Toluene may serve as a model substance for organic solvents, but is not representative for oils [25]. In 2013, Schliemann et al. used n-octane and cumene as lipophilic irritants for in vivo studies [11].

Most published studies on skin protection creams focus only on wet-work or hydrophilic irritants. There are only few historical studies investigating skin protection products against hydrophobic working materials. Additionally, the results of these studies are not promising and show inconsistent results [11,26]. While Schliermann et al. (2013) observed no measurable benefit of BC application in a double-blind, randomized trial using n-octane and cumene as model irritants, Winkler et al. (2008) in contrast, reported beneficial effects under real-life occupational conditions, especially when skin protection was combined with skin care [11,13]. However, more recent studies investigating skin protection products against hydrophobic working materials are lacking. A Cochrane review comparing BCs and moisturizers for preventing occupational irritant hand dermatitis further highlights the need for robust clinical evidence [27].

### Challenges Regarding Efficacy Testing

Regarding the efficacy testing of BCs, one major concern is the fact that they are regulated under the EU cosmetics act (No. 1223/2009) and as such do not require specific efficacy verification for the prevention of ICD and ACD, continuing the ongoing controversy around BCs [28]. Although it is not required by law, some manufacturers nonetheless opt to perform tests such as the repetitive skin exposure method on human volunteers, they are restricted to nonhazardous irritants, which limits generalizability to occupational exposures. Further, the overall lack of standardized analyses or protocols is also attributable to the skin’s inherent heterogeneity—layered structure, appendages, vasculature, resident immunity, and microbiome—making it challenging to investigate and choose the most suitable method. In vivo human studies must rely on noninvasive surrogate endpoints whereas in vitro models lack the full complexity of physiological skin [29]. The most frequently used endpoint measurements are hydration levels and TEWL. Here, the EEMCO guidelines, which were first released in 1997 and updated in 2001 and 2018, describe the different approaches and technologies available [30,31,32]. The guidelines state that these parameters can reliably detect diseased skin, as it was shown that hydration levels are low and TEWL is high due to an impaired barrier function. Even when the skin appears healthy, these results can still predict the risk of irritant contact dermatitis [32,33]. Regarding hydrophobic substances, lipids or oils may influence capacitance and might cause an occlusive effect that could decrease TEWL [34,35]. Hence, timepoints of data collection need to be carefully determined and interpreted.

Many studies usually include only one or two model irritants (e.g., sodium lauryl sulfate). From these results, researchers often claim protection against all substances of the same class. This is an overgeneralization, because hydrophilic chemicals differ in pKa, logP, and molecular size. To ensure reliable occupational safety, evaluating barrier performance across several representative substances would improve the significance of the results.

One aspect that is generally missing in most common test methods is the fact that dermal penetration and systemic uptake are not captured. Some occupational relevant substances might not cause redness or reduced cell viability in these tests but might still be able to penetrate the SC and elicit subclinical or delayed systemic effects. Accordingly, incorporating skin-permeation studies into evaluation protocols could enhance occupational safety.

The current gold standard method for investigating skin permeation is so-called Franz diffusion cells. Here, skin equivalents or ex vivo human or animal skin is clamped between two chambers—a donor and a receptor chamber. After exposing the surface of the skin to a test substance, the receptor fluid underneath can be analyzed for the amount of permeated substance. The application of a BC can be readily incorporated into this method. However, it involves constant contact of the skin or membrane with fluid, which can cause swelling and partial barrier disruption. As a result, in vitro fluxes often exceed in vivo permeation [36,37]. This and other commonly used methods are described with their respective limitations in Table 2.

In addition to methodological challenges, regulatory changes pose significant formulation challenges for industry. For example, talcum powder, long used for its absorbent properties, is likely to be prohibited in cosmetic products by 2027 due to potential carcinogenic, mutagenic and reprotoxic (CMR) classification [38]. Likewise, the restriction of PFPEs in France (2026) exemplifies how evolving regulatory landscapes necessitate reformulation and innovation in barrier cream development [39]. These regulatory changes highlight the need to align research, testing protocols, and formulation strategies to maintain product safety and efficacy.

**Table 2:** Established and frequently used methods for the efficacy testing of barrier creams.

Test Method	Model Type	Principle	Measurements	Limitations	Ref.
Patch testing	In vivo (human)	A substance is applied under a patch or chamber to a BC-treated and an untreated site for a defined duration. 24–48 h afterwards, skin irritation is determined.	TEWL, erythema (visual scoring or chromametry), skin blood flow measurements (Laser Doppler), skin hydration measurements (capacitance, conductance or impedance)	Many occupationally relevant hydrophobic substances are prohibited for human testing. Here, toxic concentrations must be avoided but irritation in the control group must be established for meaningful comparisons. While there are guidelines by the European Society of Contact Dermatitis (ESCD) available for diagnostic patch testing, none exist for the efficacy testing of BCs.	[40,41,42,43,44]
Repetitive irritation testing (RIT)	In vivo (human)	The test substance is applied repeatedly over multiple days (often up to two weeks) on BC-treated and untreated skin. The substance may be applied multiple times per day as well. Skin irritation may be assessed daily or at the end of the test phase.	TEWL, erythema (visual scoring or chromametry), skin blood flow measurements (Laser Doppler), skin hydration measurements (capacitance, conductance or impedance)	Many occupationally relevant hydrophobic substances are prohibited for human testing. Here, toxic concentrations must be avoided but irritation in the control group must be established for meaningful comparisons. No standards or guidelines are available.	[12,25]
Tape stripping	In vivo (human), ex vivo	A (hydrophobic) dye is applied onto BC-treated and untreated skin of human volunteers or excised human or animal skin. A tape is applied in a standardized manner and removed. This step is repeated with a fresh tape until a few layers of the epidermis are removed. This method investigates the depth of the penetration of the dye as well as the state of the skin. The amount of removed corneocytes correlates with skin health.	The tapes are analyzed for the content of the test substance and/or amount of corneocytes.	Dyes are only a surrogate and may not mimic real occupational substances. This model does not directly assess irritation but rather the depth of dye penetration. The standard dye for hydrophobic substances is Oil Red dissolved in ethanol. Ethanol can act as a penetration enhancer.	[45,46]
Perfused Bovine udder model	Ex vivo	The udder is removed after slaughter, and the arterial supply is cannulated and perfused with an appropriate fluid. BCs can be applied on the skin surface before exposure to a test substance. The permeated amount of the substance can be analyzed in the perfusate.	The perfusate is analyzed for concentration of the specific substance that was applied (e.g., with HPLC).	The udder must be obtained shortly after slaughter. Its skin differs from human skin (lipid composition and follicular structure). Uneven perfusion can occur due to partial clotting, vessel collapse, or tissue heterogeneity. No standards or guidelines are available.	[47,48]
Franz diffusion cells	In vitro (skin equivalents), ex vivo	A synthetic membrane, artificial skin or excised human or animal skin is clamped between two chambers of a Franz diffusion cell. The chamber underneath the skin is filled with a receptor fluid (e.g., saline) and collects the test substance which is applied on top of the dermis part of the untreated or BC-treated skin or membrane. The test substance must have adequate solubility in the receptor fluid, which is often achieved by adding ethanol or albumin in the case of hydrophobic substances. Guideline available: OECD Test Guideline 428.	The receptor fluid is analyzed for concentration of the specific substance that was applied (e.g., with HPLC).	This model investigates the permeation of substances rather than irritation. Hydrophobic substances may remain in the SC; hence, they might not be detected in the receptor fluid (there are methods available to assess the content that remained in the skin). The skin/membrane will become hyperhydrated due to constant contact with the receptor fluid during the experiment which likely increases its permeability.	[49,50,51,52]

## 4. Future Directions

In response to the inherent limitations of conventional test systems, current research increasingly emphasizes the design of more physiologically relevant and predictive models that more accurately reproduce or visualize human skin structure and barrier function [53,54,55,56,57]. While ex vivo animal tissue is a valid option to use for testing the efficacy of BCs, the ethical concerns and the heterogeneity of tissue samples warrant the need to develop and refine in vitro approaches. Here, full thickness skin equivalents (also called artificial skin) are a promising alternative to ex vivo skin. While it lacks the appendages of physiological skin, it consists of a dermis and stratified epidermis which was shown to be more comparable than monocultures of the respective cell types [58]. Current research is advancing full-thickness skin equivalents and integrating them with the mentioned test methods to derive reliable in vitro–in vivo extrapolation factors. These developments enable more standardized, higher-throughput testing—including hazardous or restricted chemicals (e.g., hydrophobic agents)—while reducing reliance on ex vivo human or animal skin. In practice, optimized skin equivalents allow systematic assessment of both hydrophilic and hydrophobic substances under controlled, reproducible conditions. Additionally, in vitro methods could be used to screen multiple irritants at the same time. To date, there is no standardized test protocol available for this application—skin irritation testing after BC use—however guidelines such as No. 439 of the Organization for Economic Cooperation and Development (OECD) termed “In Vitro Skin Irritation: Reconstructed Human Epidermis Test Method” can provide directives for new test protocols. It involves the generation of in vitro 3D epidermis models where cellular metabolism is determined after exposure to a test substance [59]. This protocol could be adapted to include the application of BCs before the exposure to known irritants. This assay can be combined with interleukin-1α measurements, giving an additional readout regarding inflammation. In addition, this method can be used with hydrophobic irritants that are prohibited or unethical to use on humans such as heptanal and trichloroethylene [60].

Furthermore, recent research aims to refine existing methods and to enable the simultaneous assessment of skin irritation and substance penetration. Here, one novel option is dermal open flow microperfusion (dOFM), where special probes with a micro-mesh area are inserted into the skin close to the surface and continuously perfused. The interstitial fluid is constantly sampled and can then be analyzed for inflammatory markers as well as the test substance [53,54]. Other new and emerging techniques for skin irritation and/or percutaneous absorption testing include Confocal Raman spectroscopy, Line-field confocal optical coherence tomography imaging and Skin-on-a-chip models [55,56,57].

Together, these approaches could significantly improve workplace safety. An overview of these novel and emerging approaches, including their key applications and limitations, is summarized in Table 3.

## 5. Conclusions

Skin protection creams can play a valuable role in preventing occupational dermatoses, particularly in situations where gloves cannot be used due to safety concerns or reduced manual dexterity. Additionally, BCs and after-work emollients can provide complementary benefits in supporting skin health under demanding workplace conditions. However, due to ongoing regulatory changes, the formulations of skin protection creams must be regularly reviewed and updated. As such, consistent and reliable testing methods are essential to ensure the continued efficacy of these products.

While clinical studies have demonstrated the effectiveness of skin protection creams against hydrophilic irritants, significant gaps remain in assessing their protective capabilities against hydrophobic substances. Ethical constraints limit the feasibility of in vivo testing, especially for hydrophobic irritants, highlighting the urgent need for innovative and standardized in vitro or ex vivo alternatives. Future research should refine in vitro and ex vivo models, the correlation between them and in vivo results, and the enhancement of workplace simulation techniques to better translate laboratory findings into real-world applications. For industry and regulators, the development of harmonized testing protocols is critical to ensure that products deliver meaningful protection in real-world occupational settings. Standardization would also facilitate cross-study comparability and inform regulatory guidance on product claims. The implications of more robust and standardized efficacy testing would also directly support workplace interventions, guide employers in selecting effective protective strategies, and ultimately reduce the incidence of occupational irritant hand dermatitis. Bridging laboratory insights with real-world conditions remains a priority for both clinicians and occupational health professionals. Therefore, a key future direction is the integration of advanced technologies to improve real-time efficacy testing and better translate laboratory results to occupational settings.

## Figures and Tables

**Table 3:** Emerging and novel methods for the efficacy testing of barrier creams.

Test Method	Model Type	Principle	Measurements	Limitations	Ref.
Reconstructed human epidermis (RHE) testing	In vitro (skin equivalents)	This method is based on lab-grown 3D keratinocyte cultures that form layers similar to a physiological epidermis. BCs can be applied before exposure to a test substance. Toxic substances that are prohibited for use in human testing can be applied in this model. No guidelines are available; however, the OECD Test Guideline 439 could be adapted to include BCs.	The converted blue formazan can be measured photometrically. Values below or equal to 50% indicate a decreased cell viability and with that, irritation. Additionally, cytokine release can be assessed as well.	This is a static model; there is a lack of appendages and immune components in the artificial epidermis.	[36,61]
Dermal open flow microperfusion (dOFM)	In vivo (human), in vitro (skin equivalents), ex vivo	Fine tubes are inserted into the dermis and connected to a peristaltic pump for perfusion. The tubes contain an open area, which allows the entry of various molecules (e.g., hydrophilic, hydrophobic, small and large molecules, cytokines, immune cells, etc.). By slowly perfusing the tubes, these molecules are transported away and collected for subsequent analysis. The system is recognized by the U.S. Food and Drug Administration (FDA) as a validated method for assessing bioequivalence of topical drug products.	The perfusate is analyzed with appropriated methods (e.g., immunoassays, HPLC, etc.).	The method is technically demanding and requires specialized equipment and specifically trained personnel. No standards or guidelines are available.	[53,54,62]
Confocal Raman Microscopy	In vivo (human), in vitro (skin equivalents), ex vivo	The specific Raman spectrum of a molecule of interest is detected by a Raman microscope directly in the skin or skin equivalent. This noninvasive method can determine the penetration depth and intensity of a molecule as well as changes in the water content and lipid order of the skin, thereby also assessing skin health simultaneously. Additionally, this method can also inform about the distribution of the compound in the skin, e.g., detecting high signal intensities around hair follicles or sweat ducts suggests follicular uptake.	Characteristic Raman spectral bands are used to detect and quantify molecules.	The detection is limited to the upper epidermis (up to 30 µm); deeper layers of the skin are not reached. The spectral bands of applied BCs could overlap with the skin or with hydrophobic test substances. The method is technically demanding and requires specialized equipment and specifically trained personnel. No standards or guidelines are available.	[63,64]
Line-field confocal optical coherence tomography	In vivo (human), in vitro (skin equivalents), ex vivo	Here, a line-shaped beam is simultaneously illuminated and detected, enabling real-time acquisition of cross-sectional and horizontal-section images of the skin. This can be utilized to measure the penetration depth of a topically applied compound as well as assess skin health.	Differences in optical backscattering between tissue structures are determined.	This method cannot assess skin health or irritation. Penetration depth is limited to 500 µm, restricting the visualization to the epidermis and upper dermis. The contrast relies on refractive-index differences, so highly reflective surface films or oily residues can cause optical artifacts. The method is technically demanding and requires specialized equipment and specifically trained personnel. No standards or guidelines are available.	[65,66]
Skin-on-a-chip model	In vitro (skin equivalents)	These microfluidic platforms integrate skin equivalents with sensors and a perusable microsystem to allow monitoring as well as continuous sampling.	The perfusate is analyzed with appropriated methods (e.g., immunoassays, HPLC, etc.). Sensors may be integrated to monitor specific parameters during testing.	Fabrication of microfluidic chips is highly complex and the inter-laboratory reproducibility is low because of custom designs and lack of standardization. Further, artificial skin lacks appendages and immune components. No standards or guidelines are available.	[67,68]

## Abbreviations

The following abbreviations are used in this manuscript:

ACD	Allergic contact dermatitis
BC	Barrier cream
CMR	carcinogenic, mutagenic and reprotoxic
dOFM	dermal open flow microperfusion
FDA	U.S. Food and Drug Administration
ICD	Irritant Contact Dermatitis
MTT	3-(4,5-Dimethylthiazol-2-yl)-2,5-Diphenyltetrazolium Bromide
OECD	Organization for Economic Cooperation and Development
PFPE	Perfluoropolyether
PPE	Personal protective equipment
SC	Stratum corneum
TEWL	Transepidermal Water Loss
